# Recurrent Chylothorax in Renal Cell Carcinoma

**DOI:** 10.7759/cureus.5196

**Published:** 2019-07-22

**Authors:** Moeezullah Beg, Hamza Arif

**Affiliations:** 1 Internal Medicine, University of Texas Health Science Center at San Antonio, San Antonio, USA; 2 Geriatric Medicine, University of Pittsburgh Medical Center St. Margaret, Pittsburgh, USA

**Keywords:** chylothorax, pleural effusion, renal cell carcinoma, malignancy associated chylothorax.

## Abstract

Chylothorax is a type of pleural effusion that results from the build up of chyle in the pleural space. Trauma and malignancies are its leading causes. Among malignancies, lymphomas cause the majority of chylothoraces. A few cases of chylothorax resulting from various solid malignancies have been reported in the literature but renal cell carcinoma (RCC) has been rarely associated. Here, we report a rare case of a unilateral chylothorax associated with a newly diagnosed RCC.

## Introduction

Chylothorax is a type of exudative pleural effusion that results from lymphatic fluid build-up in the pleural space. It can be broadly classified as traumatic and non-traumatic, with equal reported incidence [1]. Malignancies, particularly lymphomas, are the leading cause of non-traumatic chylothoraces [1-2]. A few cases of chylothorax resulting from various solid malignancies have been reported in the literature but renal cell carcinoma (RCC) has been rarely associated. Here, we present a unique occurrence of a unilateral chylothorax associated with a newly diagnosed RCC.

A part of this article was presented at the American Thoracic Society International Conference 2017 (Poster: Beg M, Arif H, Young M, Alhassan S, Bihler E. A case of recurrent chylothorax in renal cell carcinoma. American Thoracic Society 2017 International Conference; 2017: 19-24).

## Case presentation

**Figure 1:**
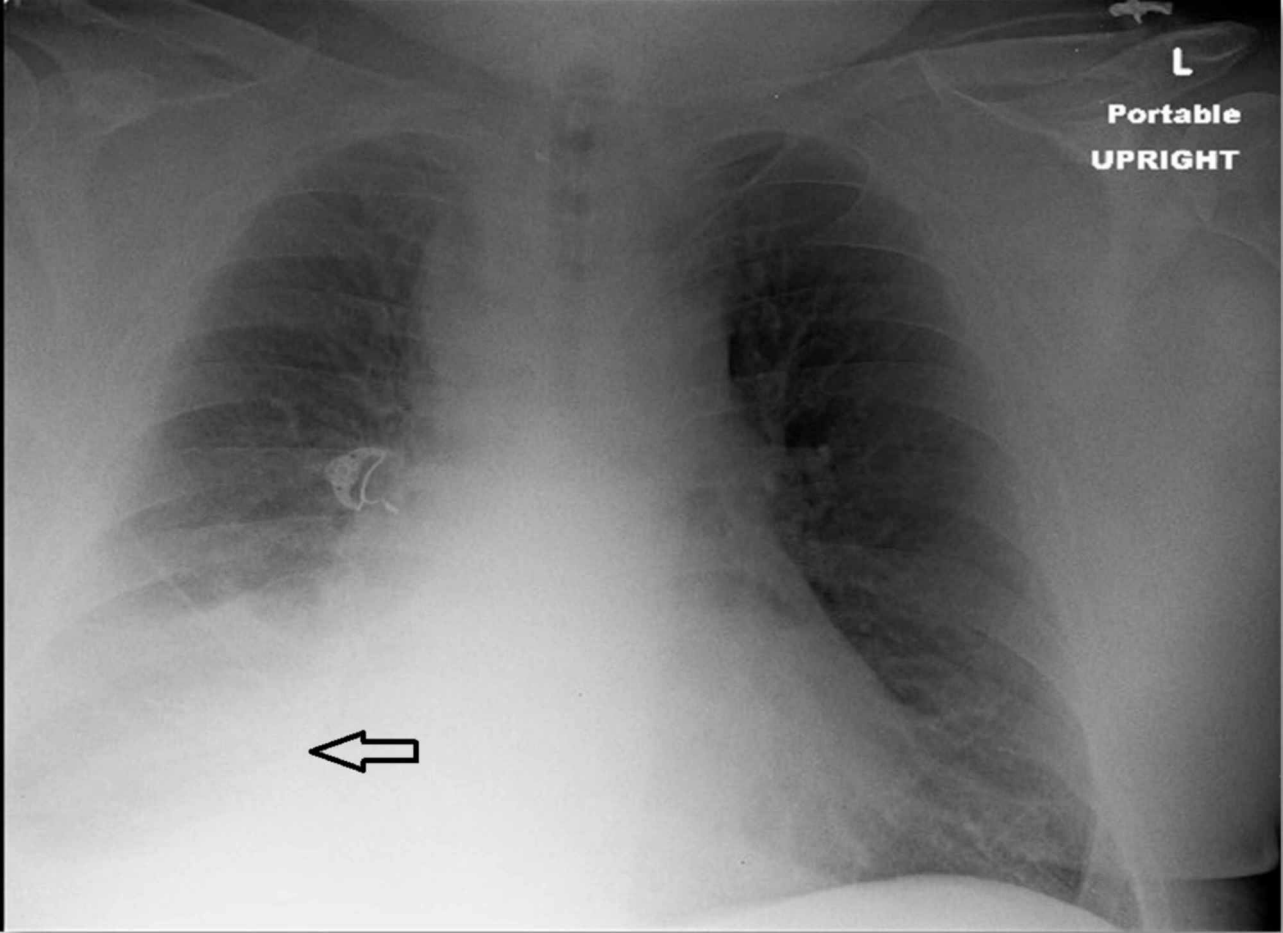
Initial chest X-ray showing right-sided pleural effusion

**Figure 2:**
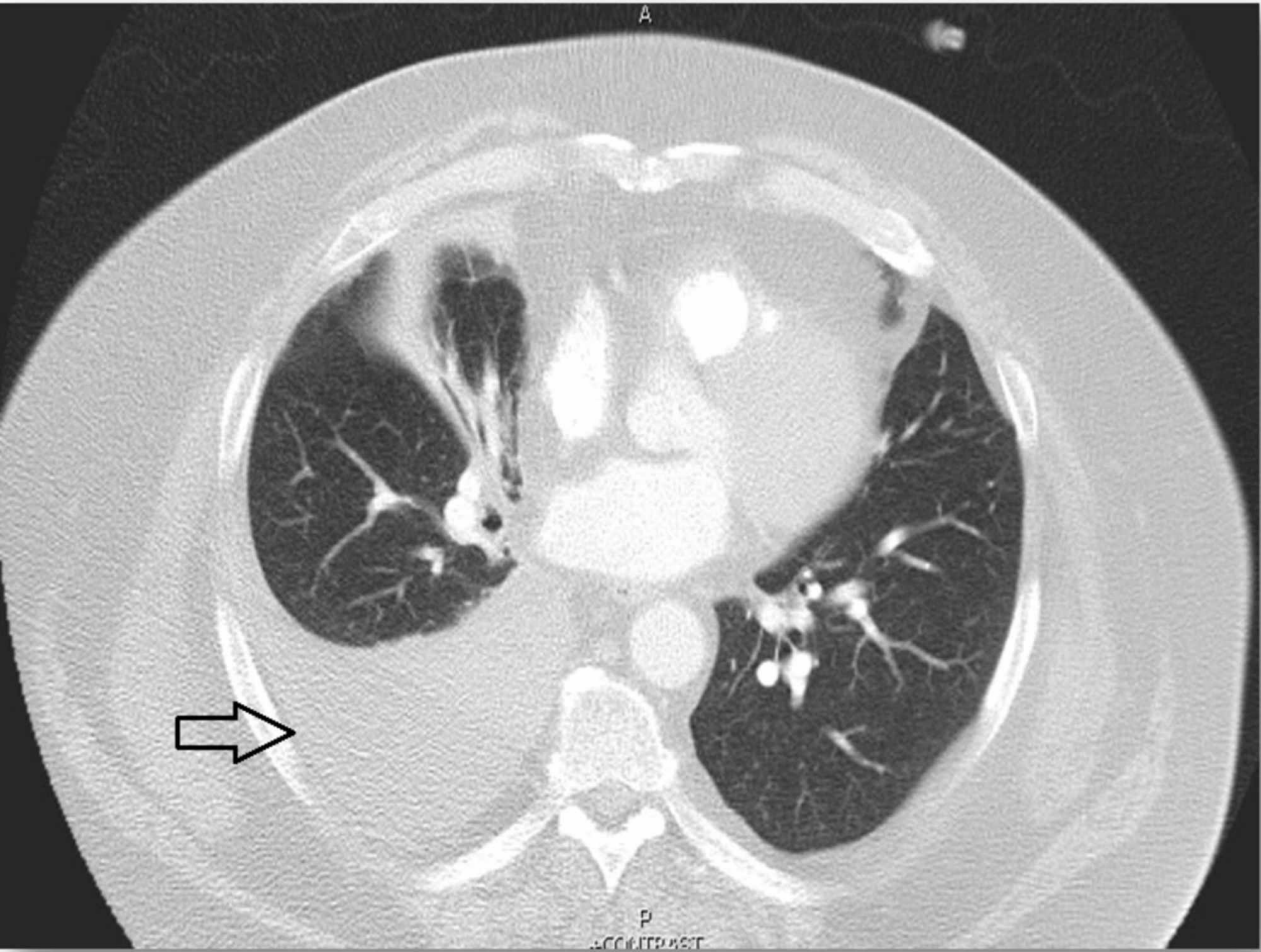
Computed tomography chest showing right-sided pleural effusion

A 61-year-old, morbidly obese gentleman presented with worsening dyspnea on exertion without any associated fever, chills, cough, or chest pain. Physical examination was unremarkable except for diminished breath sounds over the right lung. Laboratory investigations, including complete blood count and comprehensive metabolic panel, did not reveal any abnormalities. Chest radiography showed a right-sided opacity suggestive of pleural effusion (Figure 1) and computed tomography (CT) of the chest revealed a moderate-sized right-sided pleural effusion (Figure 2).

The next day, he underwent diagnostic thoracentesis followed by a small-bore chest tube. Pleural fluid analysis (Table 1) showed an exudative effusion with cholesterol of 158 mg/dl, triglycerides of 541 mg/dl, a total white blood cell (WBC) count of 3825/mcl with 96% lymphocytes predominance. Pleural fluid gram stain and culture were negative.

**Table 1:** Pleural fluid analysis LDH, lactate dehydrogenase; WBC, white blood cells.

Trait	Value
Appearance	Yellow
Protein (g/dL)	4.7
LDH (U/dL)	124
Triglycerides (mg/dL)	541
Cholesterol (mg/dL)	158
WBCs per microliter	3825
Percent lymphocytes	96%

Fluid cytology showed atypical lymphocytes and reactive mesothelial cells, and flow cytometry of the pleural fluid did not show any immunophenotypic findings diagnostic of granulocytic sarcoma or non-Hodgkin’s lymphoma.

**Figure 3:**
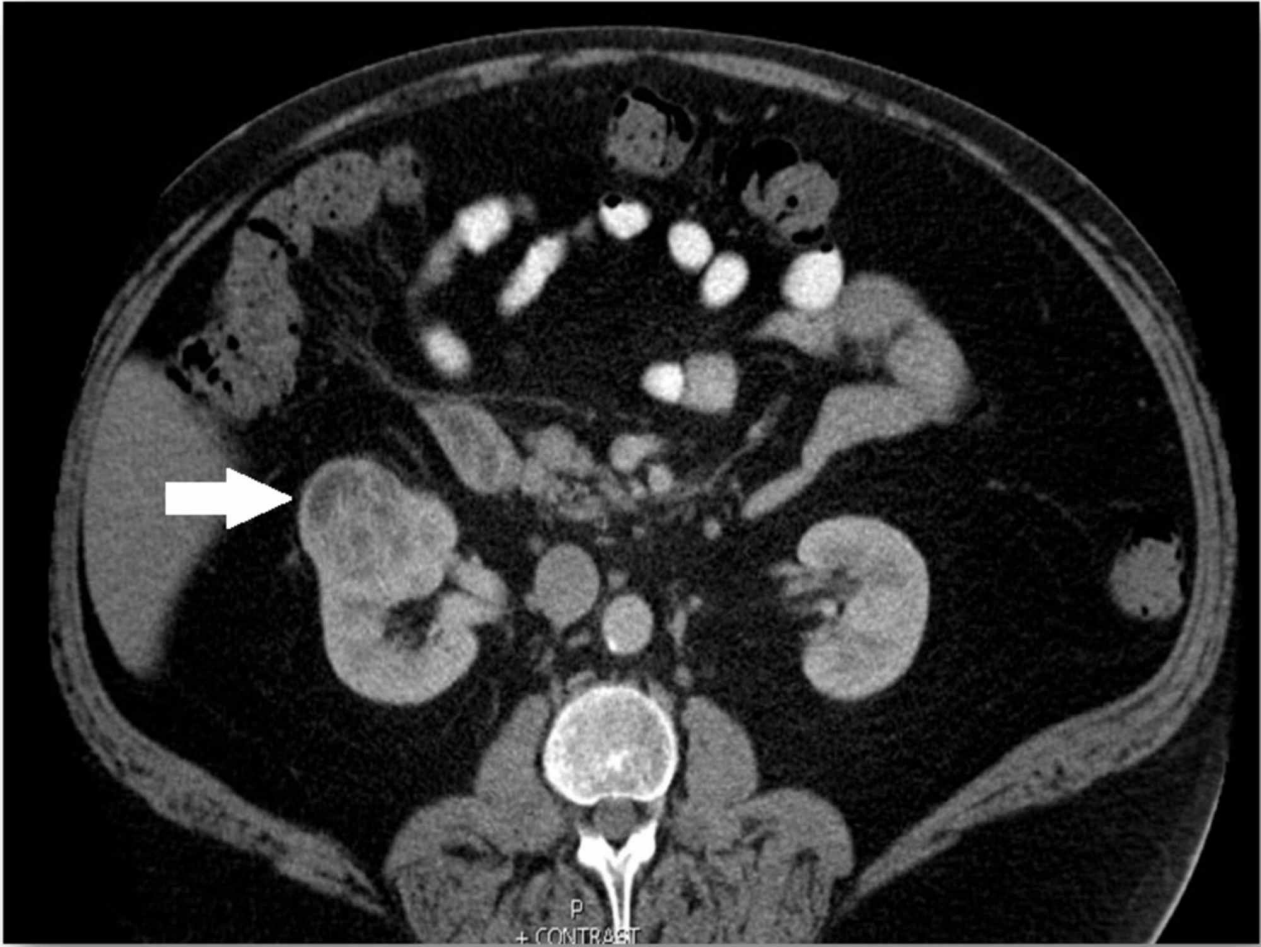
Computed tomography abdomen showing an exophytic mass originating from upper lateral aspect of the right kidney

Despite conservative medical management with a no-fat diet and therapeutic thoracentesis, the chylothorax recurred and he returned to the hospital five days later with complaints of progressively worsening shortness of breath. Repeat chest radiography showed findings consistent with recurrent right pleural effusions. Upon further investigation, a solid, enhancing, 4.9 cm right renal mass without associated lymphadenopathy was discovered on CT of the abdomen (Figure 3).

RCC was diagnosed with a biopsy and was treated with microwave ablation, as the patient was a poor surgical candidate. This led to the resolution of his recurrent chylothorax.

## Discussion

Chylothorax is characterized by the presence of chyle in the pleural space. Chyle is the lymphatic fluid primarily composed of triglycerides absorbed from the gastrointestinal tract. It is carried from the cisterna chyli in the abdomen to the central venous circulation by the thoracic duct [1]. Any disruption in the function or structure of the thoracic duct can lead to chylothorax.

Chyle is a milky-appearing fluid, which is usually exudative in character, but it may be transudative in up to 10% of cases [2]. The pleural fluid triglyceride level is a useful test for diagnostic purposes, as a level greater than 110 mg/dl is a highly specific finding for this condition [3].

Although the diagnosis of chylothorax is usually not difficult, determining its etiology can be a challenge. Broadly, chylothorax can be classified as traumatic and non-traumatic. Malignancy is the most commonly described disorder for non-traumatic causes [4] with lymphomas accounting for approximately 70% of cases [5]. Approximately 5% to 10% of cases are idiopathic, but in most of these cases, an occult neoplasm is found to be the culprit eventually [6].

No cases in association with RCC have been reported in the literature and to the best of our knowledge, this is the first reported case of chylothorax secondary to RCC. We theorize that the compression of the thoracic duct below T5 by the right-sided RCC resulted in the formation of the chylothorax in our patient. This is further supported by the fact that after microwave ablation of the RCC in our patient, the chylothorax did not recur.

## Conclusions

In some patients, chylothorax may be the initial presentation of an occult malignancy. Therefore, it is critical that all patients presenting with chylothorax and no history of trauma or recent surgery should undergo extensive work-up to look for the underlying cause, as it may be the first manifestation of an underlying malignancy as seen in this case.
